# New Quinone Antibiotics against Methicillin-Resistant *S. aureus*

**DOI:** 10.3390/antibiotics10060614

**Published:** 2021-05-21

**Authors:** Javier Campanini-Salinas, Juan Andrades-Lagos, Nicolás Hinojosa, Fabián Moreno, Pedro Alarcón, Gerardo González-Rocha, Ian E. Burbulis, David Vásquez-Velásquez

**Affiliations:** 1 Drug Development Laboratory, Faculty of Chemical and Pharmaceutical, Sciences, Universidad de Chile, Sergio Livingstone 1007, Santiago 8380492, Chile; jandrades@ug.uchile.cl (J.A.-L.); nicolas.hinojosa.torres@gmail.com (N.H.); fmorenoruz8@gmail.com (F.M.); 2 Facultad de Medicina y Ciencia, Universidad San Sebastián, Lago Panguipulli 1390, Puerto Montt 5501842, Chile; 3 Agents of Bacterial Meningitis Laboratory, Instituto de Salud Pública de Chile, Santiago 7780050, Chile; palarcon@ispch.cl; 4 Laboratorio de Investigación en Agentes Antibacterianos (LIAA), Departamento de Microbiología, Facultad de Ciencias Biológicas, Universidad de Concepción, Concepción 4070386, Chile; ggonzal@udec.cl; 5 Millennium Initiative for Collaborative Research on Bacterial Resistance (MICROB-R), Av. Las Condes 12.438, Lo Barnechea, Región Metropolitana, Santiago 8320000, Chile; 6 Centro de Investigación Biomédica, Facultad de Medicina y Ciencias, Universidad San Sebastián, Sede de la Patagonia, Lago Panguipulli 1390, Puerto Montt 5501842, Chile; ian.burbulis@uss.cl; 7 Department of Biochemistry and Molecular Genetics, University of Virginia School of Medicine, 1340 Jefferson Park Ave., Charlottesville, VA 22908, USA

**Keywords:** methicillin-resistant *S. aureus* (MRSA), drug discovery, quinone-antibiotics

## Abstract

There is an urgent need for the development of new antibiotics. Here, we describe the inhibitory activity of new quinone compounds against methicillin-resistant *Staphylococcus aureus* (ATCC^®^ 43300), methicillin-sensitive *S*. *aureus* (ATCC^®^ 29213), and two clinical isolates from Chile (ISP-213 and ISP-214). We observed 99.9% reduction in viability within 2 h of exposure without the cultures exhibiting any post-antibiotic effect, which was twice the kinetics to that observed with vancomycin. These clinical isolates did not acquire resistance to these quinone derivatives during the course of our study. We found that these compounds protected larvae of the greater wax moth, sp. *Galleria mellonella*, from infection by these MRSA clinical strains as effectively as vancomycin. These quinone derivatives are potential drug candidates worth further development.

## 1. Introduction

The World Health Organization (WHO) identified antimicrobial resistance as one of the main threats to global public health in 2019 [1]. Antimicrobial resistance has reached all continents and currently negatively impacts most countries of global health systems [2]. One of the most problematic pathogens is methicillin-resistant *Staphylococcus aureus* (MRSA). This agent causes high morbidity in the United States [3], and the Pan American Health Organization (PAHO) warns that there is a 90% prevalence of MRSA in 21 countries of the Americas [4], which indicates that patients infected with MRSA are now 64% more likely to die due to treatment failure compared to an MSSA infection [4]. Not surprisingly, the WHO generated a list of priority pathogens to focus the development of new antibacterial drugs against in 2017; MRSA ranked as a high priority [4]. Unfortunately, the development of new antibacterial drugs has progressively declined since the 1980s [5]. An analysis of new antibiotics approvals in the recent decades indicates a sharp fall in the last 30 years [6].

In particular, there has been widespread reluctance in the commercial sectors to develop new antibacterial drugs for reasons that include funding and barriers to market entry [6]. One path to obtaining new, potent activities while mitigating the costs of translation is to structurally modify drugs previously introduced to the market or drugs whose mode of action is already known [7]. Numerous governmental and non-governmental organizations support this approach with tax and social initiatives encouraging new drug development, but commercial innovation lags behind [8,9].

Compounds containing the quinone ring structure are renowned for antimicrobial and antitumoral activities [10,11,12,13]. Despite having numerous naturally-occurring and synthetic quinone derivatives being used clinically and several studies since the 1950s, the full-spectrum of antimicrobial actions associated with derivatives of this core structure is unknown. To investigate the possibility of creating new drugs with anti-MRSA activity we synthesized 17 substituted derivatives starting from a thiophenyl quinone core backbone [14]. These entirely-new derivatives differed from each other by the chemical groups added and the carbon position in which the groups were attached to the thiophenyl ring. Several compounds inhibited the growth of multi-drug resistant *Staphylococcus aureus* and *Enterococcus faecium* (minimum inhibitory concentrations of 1 to 32 μg/mL) without exhibiting toxicity to mammalian cells [14]. We found that the size and lipophilicity of the chemical groups attached to the phenyl ring modified the antimicrobial activity, with substituents in the ortho- and para-position exhibiting the greatest inhibitory activity against *S. aureus* and *E. faecium* [14].

Our preliminary findings indicated that some of these quinone derivatives might have potential use as new antibiotics but more detailed knowledge about antimicrobial activity is needed to prioritize which derivatives are most promising. For example, we do not yet know the kinetics of bactericidal/bacteriostatic activity or any information about potential post-antibiotic effects (PAEs) MRSA stains might exhibit after exposure. How easily clinical isolates might develop resistance or whether these compounds inhibit the growth of MRSA in live animals are open questions.

Here, we prioritized which derivatives to study further. We excluded derivatives that were not sufficiently soluble in water because these characteristics will not be suitable for drug development. We focused on two soluble derivatives containing either a chlorine or bromine group at the para-position to the carbon-sulfur bond linking the phenyl ring to the quinone ring [14]. These two compounds exhibited minimum inhibitory concentrations of 1 to 4 μg/mL against *S. aureus*, which was close to that observed for vancomycin at 1 μg/mL.

With the objective of verifying if these two quinone derivatives have potential to be further developed into drug candidates, we assayed therapeutic potential in vitro and in vivo. For this investigation, we refer to the chlorinated and brominated derivatives as quinone compound #1 (QNC1) and quinone compound #2 (QNC2), respectively (Supplementary Figure S1 [14]). First, we assayed the time necessary for these compounds to kill different clinical isolates of MRSA. Additionally, we evaluated if the derivatives had any post-antibiotic effects and if the MRSA isolates acquired resistance under controlled conditions.

We used MRSA-infected larvae of the greater wax moth, sp. *Galleria mellonella*, as a low-cost, rapid-use tool to assay antimicrobial dose responses in a living animal. This unusual system is easy to use and correlates well with murine models to provide an ethical alternative to testing drugs in mammals [15,16,17,18,19,20]. The larvae are susceptible to infection by a wide-range of pathogenic bacteria. In this system, larvae are infected and replicate samples are treated with a dilution series of drug treatments. After a specified period of time, disease free larvae are scored to quantify dose-response, if any. This model system does not require sophisticated equipment and has been used by various authors to test drug efficacy of similar antibacterial agents [15,16,17,18,19,20]. Herein, we present the results of these experiments and discuss the significance of our findings.

## 2. Results

### 2.1. Kill Kinetics

**Figure 1:**
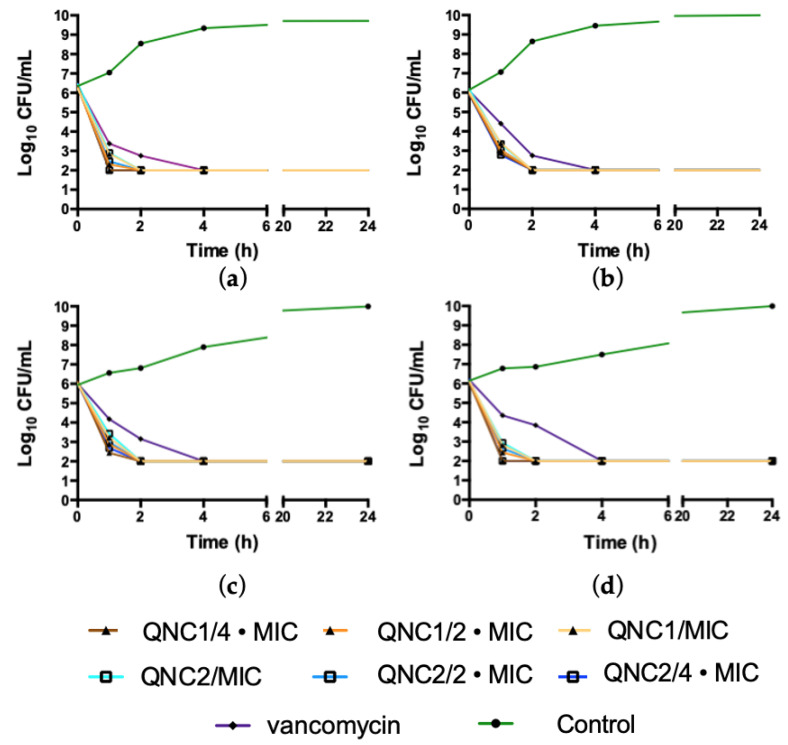
Time-Kill curves of QNC1 and QNC2 at MIC, 2 • MIC and 4 • MIC against; (**a**) MRSA ATCC^®^ 43300; (**b**) MSSA ATCC^®^ 29213; (**c**) MRSA Clinical isolates ISP-213; (**d**) MRSA clinical isolates ISP-214. Vancomycin was used for comparison.

To begin our examination of the therapeutic potential of our selected quinone derivatives, we measured microbicidal kinetics against different MRSA isolates exposed to QNC1 or QNC2. In these studies, we considered it relevant to know when 99.9% of the population studied had been killed. The mean time needed to kill 99.9% MRSA strains upon exposure to any concentration of QNC1 was 2 ± 0.17 hours (mean ± standard deviation) regardless of concentration (Figure 1). The viability of all strains exposed to vancomycin reduced 99.9% at 4 hours of exposure (Figure 1). We observed similar results for the QNC2 compound, in which 99.9% of the MRSA bacterial population was killed by 2 ± 0.17 hours exposure at all concentrations studied (Figure 1). No differences were observed in the bactericidal effects of QNC1 or QNC2 on the clinical isolates (MRSA ISP-213/ISP-214) and or the MRSA and MSSA ATCC^®^ strains tested. We observed no significant differences (*p* < 0.05) between the killing of MRSA strain by QNC1 and that of QNC2 at all time-points and concentrations studied.

### 2.2. Post-Antibiotic Effect

Continuing with our investigation of the antibacterial activity of the compounds, we determined if QNC1 or QNC2 promoted any post-antibiotic effects (PAE) in the MRSA isolates after a brief exposure to the quinone derivatives. We treated different isolates with a dilution series of QNC1 or QNC2 for 1 hour. After this time, we diluted the cultures 1000-fold to eliminate antibiotic according to previous techniques reported by Craig et al. [21]. We measured the growth curves of cultures exposed to the quinone derivatives and compared these curves to matched controlled cultures that were not treated.

**Figure 2:**
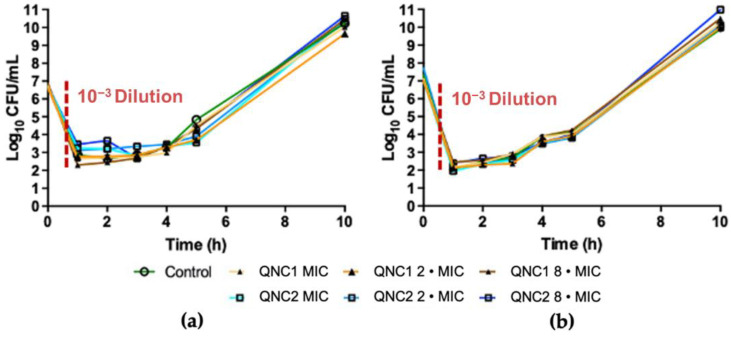
Post-Antibiotic Effects of QNC1 and QNC2 at MIC, 2 • MIC, 8 • MIC against; (**a**) MRSA ATCC^®^ 43300; (**b**) MRSA clinical isolates ISP-213.

We exposed MRSA strains to dilutions of QNC1 and QNC2 at the MIC, 2-times the MIC (2 • MIC) or 8-times the MIC (8 • MIC) for 30 min prior to removal of the drug by 1:1000 dilution (Figure 2). The ability to suppress the growth of MRSA (strain ATCC^®^ 43,300) after removal of QNC1 was 0.5 ± 0.1 hours at the MIC, 0.5 ± 0.2 hours at 2-times the MIC and 0.5 ± 0.2 hours at 8-times the MIC. The growth of MRSA (strain ATCC^®^ 43,300) was delayed by 0.5 ± 0.1 hours at the MIC, 0.5 ± 0.1 hours at 2-times the MIC and 0.75 ± 0.1 h at 8-times the MIC after exposure to QNC2 for 30 min. We observed that the MRSA clinical isolate (ISP-123) exhibited a similar PAE in response to QNC1 exposure with a growth delay of 0.5 ± 0.0 hours at the MIC, 0.5 ± 0.1 hours at 2-times the MIC and 0.5 ± 0.1 hours at 8-times the MIC. Neither quinone compound exhibited significant differences in the PAE calculated for the MRSA clinical isolate ISP-123 (*p* < 0.05). In summary, we did not observe any significant PAEs for QNC1 or QNC2 on any of the MRSA isolates tested according to the definition reported by Craig et al. [21].

### 2.3. Resistance Studies

**Figure 3:**
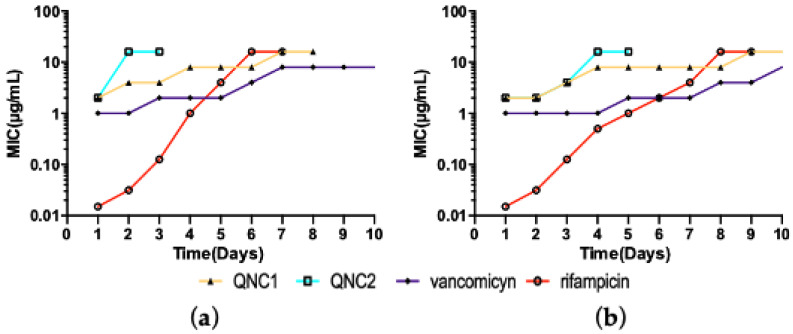
Daily changes in MIC during serial passage assay of QNC1 and QNC2 against (**a**) MSSA ATCC^®^ 29213; (**b**) MRSA clinical isolates ISP-213. Rifampicin and vancomycin were used as controls.

The potential for clinical isolates to acquire resistance to an antimicrobial drug is a critical assessment of therapeutic potential. Here, we assayed for whether the clinical MRSA or control MSSA strains acquired resistance against QNC1 or QNC2 in culture. This serial-passage test consisted of successively exposing an isolate to different concentrations of the compound and measuring changes in the MIC necessary to inhibit growth. In this case, MSSA ATCC^®^ 29213 propagated for 7 passages with the compound QNC1 increased the MIC 8 fold (Figure 3) whereas the same strain acquired an 8-fold increase in the MIC of QNC2 in only 2 passages. There were significant differences (*p* > 0.05) between the bacterial population of MSSA exposed to QNC1 versus QNC2; QNC1 selected non-susceptible MSSA populations 3.5 times faster than QNC2. The MIC of QNC1 required to kill MRSA clinical isolate ISP-213 increased 8-fold when serially passaged in the presence of drug for 9 days, while QNC2 induced the same effect in only 4 days. As in MSSA, significant differences (*p* > 0.05) between treatments were observed using the MRSA clinical isolates. We found that QNC1 promoted non-susceptible MRSA populations 2.25-times faster than QNC2. These effects were similar to that observed with vancomycin, but constituted a minor percentage of resistance acquired by the strains to the drug rifampicin (Figure 3). 

### 2.4. Effectiveness In Vivo

To investigate the antimicrobial activities of our quinone derivatives, we propagated strains of *S. aureus* in larvae of *G. mellonella* to establish a model based on previously published work [15,16,17,18,19,20]. We used the MSSA strain (ATCC^®^ 29213) and MRSA Clinical isolates ISP-213 to prepare infection model. Different inoculums were studied as reported by Desbois et al. [15]. We established the stability and safety profiles of various vehicles on *G. mellonella* larvae for this study (Table S1). We tested different formulations of the compounds QNC1 and QNC2 to validate solubility in vehicle for further administration to the larvae (Table S1). We selected glycerol because it solubilizes highly lipophilic compounds [22] and exhibited negligible negative effects on the samples or larvae. Glycerol may be combined with water in different proportions to optimize solubility of test compounds [23]. This vehicle has been previously reported to exhibit low toxicity in *G. mellonella* larvae [24]. We found that glycerol alone did not affect the susceptibility of MSSA ATCC 29213 to QNC1 or QNC1 (Table S2). We observed a similar MIC (1 to 4 μg/mL) in the presence of glycerol for both quinone compounds as compared to that previously reported by Campanini et al. [14]. We selected a 30:70 glycerol:water vehicle because of its good stability and safety on injected larvae and drug solubility (Table S1). 

**Figure 4:**
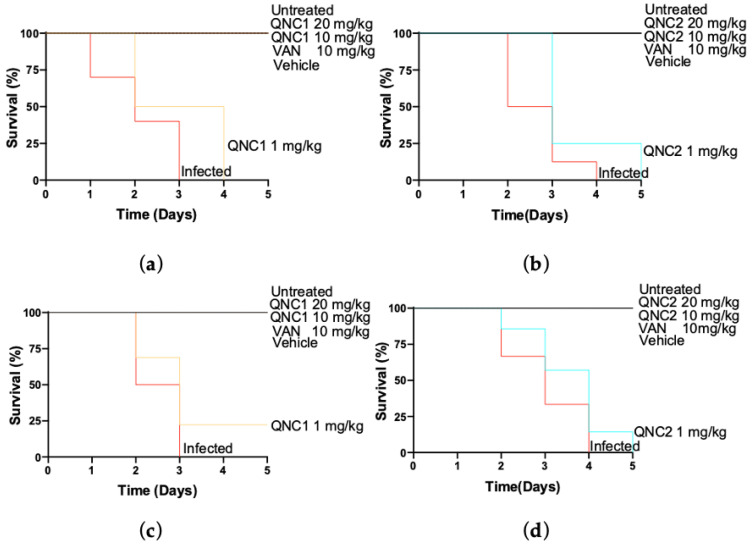
Effects of drug treatment on *G. mellonella* larvae survival inoculated with MRSA ATCC 29213 (**a**,**b**) or MRSA ISP-213 (**c**,**d**), then QNC1(**a**,**c**) or QNC2 (**b**,**d**) at 1, 10 and 20 mg/kg were administered at 0 Days (i.e., 1 hour after inoculation). Vancomycin (1 mg/kg) was used as control.

We tested the efficacy of QNC1 and QNC2 to protect *G. mellonella* larvae from infection with MRSA strain ATCC 29213 and MRSA clinical isolate ISP-213. We found that QNC1 and QNC2 had no toxic effects on larvae compared with vehicle-treated controls (Table S1 and Figure 4). The MRSA strain ATCC 29213 and MRSA clinical isolate ISP-213 aggressively infected and killed all larvae within 3 to 4 days (Figure 4). We observed that a 1 mg/kg dose of QNC1 or QNC2 did not protect the larvae from succumbing to infection by either MRSA strain (Figure 4). Higher doses of both compounds (10 and 20 mg/kg) protected larvae with 100% survival during the course of the experiment (Figure 4). We observed no difference in survival of larvae treated with QNC1 or QNC2 compared to infected larvae treated with vancomycin (10 mg/kg) (*p* < 0.05). In these groups, we observed 100% larvae survival as of the fifth day after inoculation with the MRSA strains (Figure 4).

Figure 4 shows the results using MRSA ISP-213 clinical strains for larvae inoculation. We observed no significant differences (*p* < 0.05) in effect when comparing the dose of 10 mg/kg of QNC1 or QNC2 to control groups of larvae treated with vancomycin. We observed 100% larvae survival in observed larvae groups receiving doses of 20 and 10 mg/kg QNC1 or QNC2.

## 3. Discussion

We explored the therapeutic potential of new chlorinated and brominated quinone derivatives to address the urgent need for antimicrobial drugs against multidrug-resistant MRSA [25,26]. First, we studied the kinetics of bactericidal effects on MRSA and MSSA strains exposed to different concentrations of QNC1 or QNC2. This level of bactericidal activity was consistent with other quinone derivatives we previously synthesized [14] and is similar to the Minimum Bactericidal Concentration/Minimum Inhibitory Concentration (MBC/MIC) ratio used against clinical isolates [14]. In a previously reported study, we showed that these compounds had a MBC/MIC ratio of less than 2 on MRSA isolated in Chile [14]. As defined by Craig et al., we concluded these compounds exhibited bactericidal activity on the MRSA isolates tested.

It was relevant to compare bactericidal kinetics of these compounds to a commercial antibiotic, such as vancomycin. Both quinone derivatives reduced 99.9% of the different bacterial populations studied 2 times faster than vancomycin. This result represents a comparative advantage over vancomycin since various authors have pointed out that one of the main limitations of vancomycin in the management of infections complicated by MRSA is its slow bactericidal capacity [27,28].

Microorganisms briefly exposed to inhibitory concentrations of antibiotics sometimes exhibit growth inhibition that persists even after the drug is removed. This post-antibiotic effect phenomenon has implications for dosing regimens and the determination of clinical efficacy. For this reason, it was important to identify any PAEs during the characterization stage of these new antibiotics. The PAE has traditionally been measured by exposing bacterial isolates to different drug concentrations for a short period of time, then the antibiotic is removed, and the kinetics of recovery versus the same isolate without treatment is determined. Our findings indicated that QNC1 and QNC2 promoted few, if any, PAEs upon MRSA clinical isolates. This observation is consistent with compounds that have a time-dependent antibacterial mode of action (T/MIC) and indicates that the antibacterial activity will depend on the time in which its drug concentration exceeds the MIC in plasma and not necessarily when the plasma concentration reached [29]. Antibiotics that act in a time-dependent manner (T/MIC) show a greater clinical response, with values greater than 40% of the MIC between two doses; an example is beta-lactam antibiotics that do not show PAE in Gram-negative bacilli [30]. Our results may guide concentrations’ compound administered in a murine model of infection since this type of compound (T/MIC) must be administered in repeated doses and at concentrations that do not exceed four times the MIC of the target microorganism being studied [21]. These dosage schemes would decrease the possibility of selecting resistant bacterial populations, according to recommendations by Craig W. [31].

With the advancement of antimicrobial resistance, it is necessary to study the new antibiotic potential to select for non-sensitive mutants’ populations [32]. In a best-case-scenario, newly-developed inhibitors should be difficult to inactivate through acquisition of new mutations, and in general, microorganisms should remain sensitive to the drug over time. These studies are an important part of characterizing the inhibitory activity of newly developed antimicrobial chemicals. These standard methods of assessing resistance have been in practice for decades. A common tactic is to test if a microorganism becomes less sensitive to a drug is to measure the MIC of the microorganism to the drug through a series of successive subcultures in the presence of the compound to be studied. This serial passage test is a good approximation of whether a certain compound can enrich for non-susceptible strains.

In this work, we observed substantial differences between both derivatives. QNC1 was select non-susceptible *S. aureus* populations with a rate similar to rifampin [33]. QNC2 exhibited similar kinetics to vancomycin and a slower selection speed non-susceptible populations compared to QNC1 [34]. Molecular studies are necessary to explain these differences, which theoretically could have the same mechanism of action [14]. However, both compounds have different physicochemical properties due to substitutions on the thiophenyl ring [14]. These differences might influence the stability of the compounds or access to bacterial cells. We conclude that our evidence indicates that MRSA strains acquired resistance to these derivatives no faster than to vancomycin under these conditions.

Finally, a model of infection in the larvae of *G. mellonella* was used to evaluate the effectiveness of new anti-staphylococcal agents in vivo [15]. *Galleria mellonella* has characteristics that allow it to simulate a human physiological environment; it can live at 37 °C for days with controlled humidity [35]. This insect possesses an immune system that works through hemocyte-mediated cellular responses [36] and humoral responses using antimicrobial peptides, melanin and other effector proteins [37]. These characteristics made *G. mellonela* a robust model for us to approximate the effectiveness of our compounds in vivo. We showed that a 10 mg/kg dose of QNC1 or QNC2 was as effective compared to 10 mg/kg of vancomycin in preventing the death of *G. mellonella* due to staphylococcal infection. No significant differences were found in the effectiveness observed between both derivatives. A direct relationship was observed between the administered dose of QNC1 or QNC2 and the survival percentage observed on the fifth-day post-inoculation of *S. aureus*. This collective evidence indicates these quinone compounds might represent new alternatives to today’s limited antibiotic options against MRSA.

## 4. Materials and Methods

### 4.1. Bacterial Strains and Isolates

We tested the following strains: methicillin-resistant *Staphylococcus aureus* (MRSA) ATCC^®^ 43300 and methicillin-susceptible *Staphylococcus aureus* (MSSA) ATCC^®^ 29213. We also tested the MRSA clinical isolates, ISP-213 and ISP-214, which were provided by the Chilean Public Health Institute. These MRSA strains were originally isolated from the bloodstream of septic patients and shown to be resistant to at least three additional classes of antimicrobial agents. These isolated strains were defined as multidrug-resistant. Isolates were collected in 2014 and were obtained from Chilean hospitals. All strains tested were transferred until they reached a logarithmic phase of growth.

### 4.2. Media and Antibacterial Compounds

Mueller-Hinton broth (Difco, Beckton Dickinson, Sparks, MD, USA) was used for all experiments, including killing curve, postantibiotic effect determination, and serial passage assay. Mueller-Hinton agar (Difco, Beckton Dickinson, Sparks, MD, USA) was used for performing colony counts and detecting antibiotic resistance. QNC1 and QNC2 were provided by the Drug Development Laboratory, University of Chile. Obtaining and characterization procedure was described by Campanini-Salinas et al. in 2018 [14]. Vancomycin was used as a comparator for all experiments. This was supplied by Abcam-Biochemicals^®^, Cambridge, UK. Glycerol 1 (Ensure^®^, Merck, Darmstadt, Germany) was used for vehicle formulation at in vivo tests.

### 4.3. In Vitro Activity Characterization

#### 4.3.1. Kill Kinetics

QNC1 and QNC2 were studied at 1-, 2-, or 4-times the MIC. Time–kill curves were performed in an Erlenmeyer flask with a screw cap containing Mueller-Hinton broth, using an inoculum of 5 × 10^6^–1 × 10^7^ CFU/mL in the presence of QNC1 or QNC2. Surviving bacteria were counted after 0, 1, 2, 4, 8, 12, and 24 h of incubation at 37 °C by subculturing serial dilutions of samples on Mueller Hinton agar plates. An aliquot was diluted 1000 times to remove the tested compounds [21]. 100 uL was transferred to a Mueller-Hinton agar plate, and the inoculum was dispersed with a Drigalsky loop previously sterilized with heat and 75% ethanol. A bactericidal effect was defined as a ≥3 log10 CFU/mL decrease compared with the initial inoculum after 24 h of incubation [21]. A flask inoculated with MH broth with no antibiotic served as sterility control. Vancomycin was used as control. Tests were carried out in triplicate.

#### 4.3.2. Post-Antibiotic Effect Determination

PAE method was used as described by Mercier et al. [38]. QNC1, QNC2, and vancomycin were tested at different multiples of the MIC. Mueller-Hinton I broth was used in the test tubes, and a 1/1000 dilution removed the antibiotics after the bacteria were exposed to them for 1 h. The PAE was calculated by using Equation (1):

PAE = *T* − *C* (1)

where *T* is the time required for the count in the test culture to increase 1 log10 above the count observed immediately after drug removal, and *C* is the time required for the count of the untreated control tube to increase by 1 log10.

#### 4.3.3. Serial Passage Assay

The serial passage method was used as described by Silverman et al. [39]. Briefly, on the first day, Mueller-Hinton broth containing QNC1 or QNC2 at 0.25-, 0.5-, 1-, 2-, or 4-times the MIC was inoculated with different MRSA strains from a single colony. Cultures were incubated overnight at 37 °C with shaking. From the highest concentration that supported growth, cultures were diluted 1:10,000 into fresh media plus daptomycin at two-fold dilutions. This process was continued for 21 days or until three successive cultures failed to show any decrease in susceptibility. The transfers were carried out until there was growth in all the cultures. Cultures that showed growth were subcultured in drug-free HAM plates, and MIC was determined by the method of microdilution following the CLSI protocol after 24 h [40]. After the test, the 4× CIM concentration tubes with bacterial growth were again seeded in compound-free MHA plates, and subsequently, 3 passages were made in compound-free MHA plates, determining the MIC in each passage.

### 4.4. Galleria mellonella Infection Model

*G. mellonella* larvae obtained from a commercial supplier (BioBichos, Ltd., Chillan, Chile) were used. All experiments were performed according to the protocols described by various authors [15,41,42]. Briefly, study groups consisted of 10 final stages of *G. mellonella* larvae, with an approximate weight of 250 mg each. They were randomly selected. A Hamilton^®^ syringe (Merck^®^ Ltd., Kenilworth, NJ, USA) was used to inject the larvae—inoculation volume used in each test was 10 uL. MRSA inoculum (CFU/mL) that decreases the survival of *G. mellonella* larvae was studied. Inoculum that managed to reduce survival to less than 20% in 72 h was selected. Once the inoculum of bacteria was chosen and the safety of the compounds to be tested was determined, the larvae were inoculated with the chosen suspension. After 1 h, they were injected with the compound to be studied at different concentrations. After each treatment, the larvae were incubated for 5 days at 37 °C, 80% humidity, and their survival was determined daily. One group of larvae was injected with phosphate buffer solution, and another group was not subjected to treatment. Both groups were used as controls in each of the experiments. The results of any experiment with more than two dead larvae in any control group were discarded.

### 4.5. Statistical Analysis

The data were analyzed with one-way ANOVA and t-tests, with the criterion for statistical significance set at *p* < 0.05, using the GraphPad Prism 5.03 program (GraphPad Software, Inc., San Diego, CA, USA, www.graphpad.com, accessed on 5 May 2014). Infection model data were plotted using the Kaplan–Meier method, and comparisons between groups were made using the log-rank test.

## 5. Conclusions

Herein, we demonstrated that quinone compounds, QNC1 and QNC2, are effective and safe in a model of *S. aureus* infection in *G. mellonella*. They had rapid bactericidal activity, and the QNC2 derivative has a low rate of selection of non-susceptible populations. Based on the evidence shown, we believe that the compounds have therapeutic potential and are candidates to advance towards studies in murine models.

## 6. Patents

Chilean Patent Application number 201503780, PCT/CL2016/050080; EPO 16880235.3; MX/a/2018/008192; US20190367505, CN109121411, EP3404026 titled: “Pyrimi-dine-Isoquinoline-Quinone Derived Compounds, their Salts, Isomers, Pharmaceutically Acceptable Tautomers; Pharmaceutical Composition; Preparation Procedure; and their Use in the Treatment of Bacterial and Multiresistant Bacterial Diseases.”

## Data Availability

Data is contained within the article or supplementary material.

## Supplementary Materials

The following are available online at https://www.mdpi.com/article/10.3390/antibiotics10060614/s1. Figure S1: Most potent quinonic compounds against multiresistant gram-positive isolates, Table S1: Attributes of the different vehicles to incorporate quinonic compounds, Table S2: Antibacterial activity of quinonic compounds in different vehicles.

## References

[1] White A., Hughes J.M. (2019). Critical importance of a one health approach to antimicrobial resistance. EcoHealth.

[2] O’neill J. (2014). Antimicrobial Resistance: Tackling a Crisis for the Health and Wealth of Nations.

[3] Boucher H.W., Talbot G.H., Bradley J.S., Edwards J.E., Gilbert D., Rice L.B., Scheld M., Spellberg B., Bartlett J. (2009). Bad bugs, no drugs: No ESKAPE! An update from the Infectious Diseases Society of America. Clin. Infect. Dis..

[4] World Health Organization (2014). Antimicrobial Resistance: Global Report on Surveillance.

[5] Luepke K.H., Suda K.J., Boucher H., Russo R.L., Bonney M.W., Hunt T.D., Mohr J.F. (2017). Past, present, and future of antibacterial economics: Increasing bacterial resistance, limited antibiotic pipeline, and societal implications. Pharmacotherapy.

[6] Silver L.L. (2011). Challenges of antibacterial discovery. Clin. Microbiol. Rev..

[7] Campanini-Salinas J., Andrades-Lagos J., Mella-Raipan J., Vasquez-Velasquez D. (2018). Novel classes of antibacterial drugs in clinical development, a hope in a post-antibiotic era. Clin. Microbiol. Rev..

[8] Simpkin V.L., Renwick M.J., Kelly R., Mossialos E. (2017). Incentivising innovation in antibiotic drug discovery and development: Progress, challenges and next steps. J. Antibiot..

[9] Shrivastava S.R., Shrivastava P.S., Ramasamy J. (2018). World health organization releases global priority list of antibiotic-resistant bacteria to guide research, discovery, and development of new antibiotics. J. Med. Soc..

[10] Ishidate M., Kobayashi K., Sakurai Y., Sato H., Yoshida T. (1955). Experimental Studies on Chemotherapy of Malignant Growth Employing Yoshida Sarcoma Animals XI. Effect of quinone derivatives, antibiotics, alkaloids, organoarsen, and other miscellaneous compounds. Gan.

[11] Resegotti L., Infelise V.E. (1966). The effect of a combination of phenanthroline quinone plus iodochlorhydroxyquinoline in the prevention of dysvitaminosis caused by antibiotics. Minerva Med..

[12] Wanke H., Kersten W., Kersten H. (1969). Polysomes in Bacillus subitilis: Influence of amino quinones and quinone antibiotics on the synthesis and stability of mRNA. Hoppe-Seyler’s Z. Physiol. Chem..

[13] Nagasawa T., Fukao H., Irie H., Yamada H. (1984). Sakyomicins. A, B, C and D: New quinone-type antibiotics produced by a strain of nocardia taxonomy, production, isolation and biological properties. J. Antibiot..

[14] Campanini-Salinas J., Andrades-Lagos J., González-Rocha G., Choquesillo-Lazarte D., Dragnic S.B., Faúndez M., Alarcón P., Silva F., Vidal R., Salas-Huenuleo E. (2018). A new kind of quinonic-antibiotic useful against multidrug-resistant *S. aureus* and *E. faecium* infections. Molecules.

[15] Desbois A.P., Coote P.J. (2011). Wax moth larva (*Galleria mellonella*): An in vivo model for assessing the efficacy of antistaphylococcal agents. J. Antimicrob. Chemother..

[16] Desbois A.P., Coote P.J. (2012). Utility of greater wax moth larva (*Galleria mellonella*) for evaluating the toxicity and efficacy of new antimicrobial agents. Adv. Appl. Microbiol..

[17] Tsai C.J.-Y., Loh J.M.S., Proft T. (2016). *Galleria mellonella* infection models for the study of bacterial diseases and for antimicrobial drug testing. Virulence.

[18] Cutuli M.A., Petronio G., Vergalito F., Magnifico I., Pietrangelo L., Venditti N., Di Marco R. (2019). *Galleria mellonella* as a consolidated in vivo model hosts: New developments in antibacterial strategies and novel drug testing. Virulence.

[19] Hornsey M., Wareham D.W. (2011). In vivo efficacy of glycopeptide-colistin combination therapies in a *Galleria mellonella* model of *Acinetobacter baumannii* infection. Antimicrob. Agents Chemother..

[20] Piatek M., Sheehan G., Kavanagh K. (2020). Utilising *Galleria mellonella* larvae for studying in vivo activity of conventional and novel antimicrobial agents. Pathog. Dis..

[21] Craig W.A. (1991). The postantibiotic effect. Clin. Microbiol. Newsl..

[22] Srivastava V.C. (2019). Glycerol as a Green Solvent in Organic Reactions. Mater. Res. Found..

[23] Niazi S.K. (2019). Handbook of Pharmaceutical Manufacturing Formulations: Volume Four, Semisolid Products.

[24] Allegra E., Titball R.W., Carter J., Champion O.L. (2018). *Galleria mellonella* larvae allow the discrimination of toxic and non-toxic chemicals. Chemosphere.

[25] Livermore D.M. (2004). The need for new antibiotics. Clin. Microbiol. Infect..

[26] Kmietowicz Z. (2017). Few novel antibiotics in the pipeline, WHO warns. BMJ.

[27] Sakoulas G., Moise-Broder P.A., Schentag J., Forrest A., Moellering R.C., Eliopoulos G.M. (2004). Relationship of MIC and bactericidal activity to efficacy of vancomycin for treatment of methicillin-resistant *Staphylococcus aureus* bacteremia. J. Clin. Microbiol..

[28] Kollef M.H. (2007). Limitations of vancomycin in the management of resistant staphylococcal infections. Clin. Infect. Dis..

[29] Mandell G.L., Douglas R.G., Bennett J.E. (2014). Principles and Practice of Infectious Diseases.

[30] Goodman L.S. (1996). Goodman and Gilman’s the Pharmacological Basis of Therapeutics.

[31] Craig W. (1993). Pharmacodynamics of antimicrobial agents as a basis for determining dosage regimens. Eur. J. Clin. Microbiol. Infect. Dis..

[32] Martínez J.L., Baquero F., Andersson D.I. (2011). Beyond serial passages: New methods for predicting the emergence of resistance to novel antibiotics. Curr. Opin. Pharmacol..

[33] Sakoulas G., Alder J., Thauvin-Eliopoulos C., Moellering R.C., Eliopoulos G.M. (2006). Induction of daptomycin heterogeneous susceptibility in *Staphylococcus aureus* by exposure to vancomycin. Antimicrob. Agents Chemother..

[34] Clark C., Kosowska-Shick K., McGhee P., Dewasse B., Beachel L., Appelbaum P.C. (2009). Resistance selection studies comparing the activity of razupenem (PTZ601) to vancomycin and linezolid against eight methicillin-resistant and two methicillin-susceptible *Staphylococcus aureus* strains. Antimicrob. Agents Chemother..

[35] Ramarao N., Nielsen-Leroux C., Lereclus D. (2012). The insect *Galleria mellonella* as a powerful infection model to investigate bacterial pathogenesis. J. Vis. Exp..

[36] Browne N., Heelan M., Kavanagh K. (2013). An analysis of the structural and functional similarities of insect hemocytes and mammalian phagocytes. Virulence.

[37] Harding C.R., Schroeder G.N., Collins J.W., Frankel G. (2013). Use of *Galleria mellonella* as a model organism to study Legionella pneumophila infection. J. Vis. Exp..

[38] Mercier R.-C., Houlihan H.H., Rybak M.J. (1997). Pharmacodynamic evaluation of a new glycopeptide, LY333328, and in vitro activity against *Staphylococcus aureus* and *Enterococcus faecium*. Antimicrob. Agents Chemother..

[39] Silverman J.A., Oliver N., Andrew T., Li T. (2001). Resistance studies with daptomycin. Antimicrob. Agents Chemother..

[40] CLSI (2009). Methods for Dilution Antimicrobial Susceptibility Tests for Bacteria that Grow Aerobi-Cally, Approved Standard.

[41] Luther M.K., Arvanitis M., Mylonakis E., LaPlante K.L. (2014). Activity of daptomycin or linezolid in combination with rifampin or gentamicin against biofilm-forming *Enterococcus faecalis* or *E. faecium* in an in vitro pharmacodynamic model using simulated endocardial vegetations and an in vivo survival assay using *Galleria mellonella* larvae. Antimicrob. Agents Chemother..

[42] Gibreel T.M., Upton M. (2013). Synthetic epidermicin NI01 can protect *Galleria mellonella* larvae from infection with *Staphylococcus aureus*. J. Antimicrob. Chemother..

