# Supplementary material for: New Quinone Antibiotics against Methicillin-Resistant S. aureus
Source: Antibiotics (Basel). 2021 May 21;10(6):614. doi: 10.3390/antibiotics10060614 (PMC8224091; doi:10.3390/antibiotics10060614)
Supplement: Supplementary file 1 [file antibiotics-10-00614-s001.zip › antibiotics-1163484-supplementary.pdf]

antibiotics

# New Quinone Antibiotics against Methicillin-Resistant *S. aureus*

## Supplementary Materials

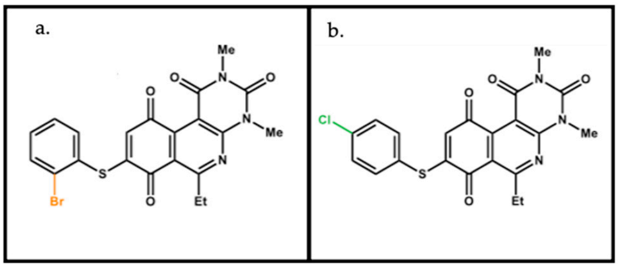


**Figure S1.** Most potent quinonic compounds against multiresistant gram-positive isolates, reported by Campanini-Salinas et al. [10]. **a**. QNC1 **b**. QNC2.

**Table S1.** Attributes of the different vehicles to incorporate quinonic compounds.

| Vehicle Name | Composition * | Stability ** | Fluidity Hamilton® Syringe | % Survival *G. Mellonella* Larvae |
|---|---|---|---|---|
| 0,9% Sodium Chloride | Distilled water<br>Sodium Chloride<br>Dimethyl sulfoxide | pp | yes | ND |
| 100% Glycerol | Glycerol | pp | not | ND |
| 60% Glycerol | Glycerol<br>Distilled water<br>Dimethyl sulfoxide | pp | not | ND |
| 50% Glycerol | Glycerol<br>Distilled water<br>Dimethyl sulfoxide | pp | not | ND |
| 40% Glycerol | Glycerol<br>Distilled water<br>Dimethyl sulfoxide | not pp | not | ND |
| **30% Glycerol** | **Glycerol<br>Distilled water<br>Dimethyl sulfoxide** | **not pp** | **yes** | **100%** |

* All vehicles contain QNC1 or QNC2; ** pp: presence of precipitate; not pp: absence of precipitate; ND: Not Determinated.

Table S1 summarizes the composition of the different vehicles used to incorporate QNC1 or QNC2. This to be administered to the *G. mellonella* larvae to test its antibiotic efficacy. First, the stability of the quinonic compounds was evaluated at 37 °C for 24 h. It was observed if after time the compounds precipitated. Then it was determined if the different formulations could flow through the Hamilton® syringe. The formulation containing 30% glycerol was selected for its good stability and its correct flow through the Hamilton syringe. Finally, it was studied whether the vehicle could affect the survival of *G. mellonela* larvae, for 5 days at 37 °C. It was evidenced that the selected vehicle did not affect the survival of the group of larvae studied (n = 10).

**Table S2.** Antibacterial activity of quinonic compounds in different vehicles.

| **Strains** | **Minimum inhibitory concentration (MIC)** | | | |
|---|---|---|---|---|
| | **QNC1** | | **QNC2** | |
| | **+Water** | **+30% Glycerol** | **+Water** | **+30% Glycerol** |
| MSSA **ATCC®29213** | 4 µg/mL | 4 µg/mL | 4 µg/mL | 4 µg/mL |

It was studied whether glycerol can affect could affect the susceptibility of MSSA ATCC 29213 to QNC1 or QNC1. The MIC of the derivatives suspended in the selected vehicle was determined. It was observed that the MIC of both derivatives was the same as previously reported by Campanini et al [10].
